# Joint effects of polycyclic aromatic hydrocarbons, smoking, and XPC polymorphisms on damage in exon 2 of KRAS gene among young coke oven workers

**DOI:** 10.3389/fpubh.2022.945955

**Published:** 2022-08-05

**Authors:** Siqin Chen, Xingyue Yin, Yuefeng He, Qinghua He, Xiaomei Li, Maosheng Yan, Suli Huang, Jiachun Lu, Binyao Yang

**Affiliations:** ^1^Innovation Center for Advanced Interdisciplinary Medicine, Guangzhou Key Laboratory of Enhanced Recovery After Abdominal Surgery, The Fifth Affiliated Hospital of Guangzhou Medical University, Guangzhou, China; ^2^Department of Environmental and Occupational Health, School of Public Health, Kunming Medical University, Kunming, China; ^3^Department of Physical Factors and Occupational Health, Guangdong Province Hospital for Occupational Disease Prevention and Treatment, Guangzhou, China; ^4^Department of Environment and Health, Shenzhen Center for Disease Control and Prevention, Shenzhen, China; ^5^The State Key Lab of Respiratory Disease, The First Affiliated Hospital, The School of Public Health, Guangzhou Medical University, Guangzhou, China

**Keywords:** polycyclic aromatic hydrocarbons, 1-hydroxypyrene, KRAS gene, XPC polymorphism, joint effect

## Abstract

Genetic polymorphisms may contribute to individual susceptibility to DNA damage induced by environmental exposure. In this study, we evaluate the effects of co-exposure to PAHs, smoking and XPC polymorphisms, alone or combined, on damage in exons. A total of 288 healthy male coke oven workers were enrolled into this study, and urinary 1-hydroxypyrene (1-OH-Pyr) was detected. Base modification in exons of KRAS and BRAF gene, and polymorphisms of XPC were determined in plasma by real-time PCR. We observed 1-OH-Pyr was positively related to damage in exon 2 of KRAS (KRAS-2) and in exon 15 of BRAF (BRAF-15), respectively, and KRAS-2 and BRAF-15 were significantly associated with increased 1-OH-Pyr. A stratified analysis found 1-OH-Pyr was significantly associated with KRAS-2 in both smokers and non-smokers, while 1-OH-Pyr was significantly associated with BRAF-15 only in smokers. Additionally, individuals carrying both rs2228001 G-allele (GG+GT) and rs3731055 GG homozygote (GG) genotype appeared to have more significant effect on KRAS-2. The high levels of 1-OH-Pyr were associated with KRAS-2 only in rs2228001 GG+GT genotype carriers and the high levels of 1-OH-Pyr were associated with KRAS-2 only in rs3731055 GG genotype carriers and the most severe KRAS-2 was observed among subjects carrying all four of the above risk factors. Our findings indicated the co-exposure effect of PAHs and smoking could increase the risk of KRAS-2 by a mechanism partly involving XPC polymorphisms.

## Introduction

Polycyclic aromatic hydrocarbons (PAHs), producing from living environment including smoking, vehicle exhaust emissions, and fuel combustion, are a group of important components in the air pollution, and attract widespread concerns in China (1, 2); PAHs existing in some occupational environments such as coke production are also measured and assessed for the early impacts on health risks (3–5). As is known to all, PAHs are a well-known mixture complex of carcinogen with toxicity and mutagenicity, and epidemiological evidence illustrated urinary 1-hydroxypyrene (1-OH-Pyr) is highly associated with the total concentration of PAH metabolites in both smokers and non-smokers (6) and several evidences suggested that urinary 1-OH-Pyr, used as a measure of total absorbed dose, could be a comprehensive biomarker of exposure to PAHs (7, 8). Therefore, urinary 1-OH-Pyr is considered as a suitable indicator to evaluate the degree of PAHs exposure due to its convenience, accessibility and effectiveness.

It has been demonstrated that PAHs exposure can lead to early deleterious alters on DNA including oxidative DNA damage (9), double-strand DNA breaks (10), reactive oxygen species generation and oxidative stress (11), genetic exon damage (12), which may accumulate genotoxic damage, change cell functions, and make people susceptible to mutagenesis and carcinogenic processes. Also, many researches have revealed that excessive exposure to PAHs may increase the risk of lung cancer (13, 14). In particular, an investigation analyzing the association between lung cancer somatic mutations and occupational exposure in never-smokers shows that patients exposed to PAHs were mostly diagnosed with gene v-raf murine sarcoma viral oncogene homolog B1 (BRAF) mutation and gene kirsten rat sarcoma viral oncogene homolog (KRAS) mutation (15). The gene KRAS is one of the RAS family members and is the most frequent mutation of them (16). More importantly, KRAS is the most frequent oncogene in non-small cell lung cancer and the lung cancers activated by KRAS mutation have serious outcomes in both early-stage and advanced metastatic settings (17, 18). More evidences revealed KRAS gene mutation, existing in the lung tumor patients, is related to the PAHs exposure from smoking and coal combustion (19, 20). Also, another research showed that particular matter 2.5 aggravates the DNA damage and apoptosis involving the upregulating of expression of p-BRAF/BRAF (21). However, the association of co-exposure to PAHs and smoking and exon damage in KRAS or BRAF gene still could contain further proof.

Epidemiologic evidence indicated that gene damage can be also regulated by genetic factors. Studies supported that single nucleotide polymorphisms (SNPs) of genes can modulate diseases and gene mutation (22, 23). The SNP of the xeroderma pigmentosum group C (XPC), which is responsible for global nucleotide excision repair, an important human DNA repair system, have been investigated that it can modulate the DNA damage level following exposure to PAHs (24). A study reveals the important role of XPC protein, which may effect the inflammation, oxidative stress, and DNA damage process in the protection against carcinogenic potential of urban air pollution (25). It is also reported that the genetic polymorphisms of XPC may predict inter-individual variation in DNA damage levels due to exposure (26). In addition, XPC is considered to help repair DNA damage induced by KRAS (27). However, the function of the polymorphisms of XPC gene on the KRAS and BRAF gene damage is still unknown.

We have previously demonstrated that individuals with FEN1 rs174538 GA+AA genotype have greater effects of urinary 1-OH-Pyr on exon damage in EGFR gene compared to those with rs174538 GG genotype after controlling for various confounders, and the statistically significant interactive effect between rs174538 genotype and urinary 1-OH-Pyr on exon damage in EGFR gene was observed (12). Nevertheless, neither the co-exposure effects of PAHs and smoking on exon damage in KRAS and BRAF nor their effects modified by XPC genetic polymorphisms had been investigated. The present analysis on whether the co-exposure effect of PAHs and smoking was involved in increasing the risk of exon damage in KRAS and BRAF gene and whether their effects were modulated to some extent by XPC genetic polymorphisms was complementary to our previously published data.

## Materials and methods

### Study Subjects

As described in our previous study (12), a total of 295 male coke oven workers were included in our investigation, aged between 19 and 35 years, from a coking plant in south region of China. The subjects excluded from the study were as follows: a) If the subjects had prior history of major diseases such as cancer; b) if they were treated with radiotherapy and chemotherapy as classical DNA damaging agents within the past 6 months; and c) if the subjects had continuous exposure in the workshop < 3 months. Each of the participants signed informed consent and filled in the occupational health questionnaire concerning demographic information, occupational history, medical history, and lifestyle including working years and smoking status and other data. Those who smoke < 1 cigarette a day are considered non-smokers; otherwise, subjects are considered smokers. Following the face-to-face interviews of questionnaires, all participants provided 20 ml of spot urine samples in 50 ml polyethylene tubes at the end of each work shift and 5 ml of venous blood in disposable ethylenediaminetet-raacetic acid anticoagulant tubes, and all samples were stored at −80°C until laboratory examinations. After excluding 2 participants with no available urinary samples and 5 with inadequate plasma sample volume, the left 288 male coke oven workers were included in this study. We then divided 288 participants into three groups according to the tertiles of 1-OH-Pyr levels: the low-, intermediate-, and high-exposure groups. This study was approved by the Ethics Committee of Guangzhou Medical University (KY01-2019-02-09), and all subjects provided written informed consent to participate in the study.

### Measurement of urinary 1-OH-Pyr and urinary creatinine

The concentration of urinary 1-OH-Pyr was detected by gas chromatography-mass spectrometry (GC/MS) as previously described (12). A 3 ml of urine was mixed with 20 μl of 1-OHP-d9 solution, 1 ml of acetate acid buffer (0.5 M, pH 5.0), and 20 μl of β-glucuronidase/sulfatase (Sigma–Aldrich, Munich, Germany) for all night at 37°C. 1.5 mg of MgSO4·7H_2_O was added to saturate the hydrolyzed urinary samples. After extracting twice using 1.5 ml of *n*-hexane and centrifugating at 300 *g* for 10 min, we used nitrogen to treat with the organic extracts, and mixed 100-μl BSTFA with the residue extractives following incubation at 90°C for 45 min. Finally, we extracted 1 μl to inject on the GC/MS system (Agilent, Santa Clara, CA). Considering the inter-individual variations in urinary metabolites on dilution status, we measured urinary creatinine concentration employing an automated clinical chemistry analyzer according to Jaffe's colorimetric method to calibrate urinary 1-OH-Pyr and expressed as micromoles per millimole of creatine.

### Determination of damage index of exon 2 of KRAS and damage index of exon 15 of BRAF

The DNA extract procedures and the amplification process have been described previously (12). We designed the primer of KRAS-2 and BRAF-15, and used β-actin as internal reference; the primers are listed as follows: β-actin (forward: CGGGAAATCGTGCGTGACAT; reverse: GAAGGAAGGCTGGA AGAGTG); exon 2 of KRAS gene (forward: GGCCTGCTGAAAATGACTGAATATAA; reverse: AAAGAATGGTCCTGCACCAGTA); exon 15 of BRAF gene (forward: TCATGAAGACCTCACAGTAAAAATAGG; reverse: AGCAGCATCTCAGGGCCAAA). We added 10-μl 2× UltraSYBR mixture, 0.8-μl primer mixture, containing 0.4 μl of 10-μM forward primer and 0.4 μl of 10-μMreverse primer, and 1-μl DNA sample into the 96-well plate, and replenished 8.2-μl H_2_O to get a 20-μl final volume. We used the real-time fluorescence quantitative PCR instrument (Roche LightCycler96, America) running the amplification process, which began with 95°C for 600 s and 45 cycles of 95°C for 10 s, 60°C for 10 s, and 72°C for 15 s. After the amplification process, we used the value of ΔCt to express the degree of damage index of the gene just as we have referred. According to the method of Sikorsky et al., the mean modified efficiency of PCR was positive correlated with 2^Ct1−*Ct*0^, where Ct1 means the target genes and Ct0 means the internal genes (28). In this study, we used 2^Ct1−*Ct*0^ to represent of the damage index of gene.

### Genotyping examination and high-resolution melting (HRM) analysis

To further determine and analyze the genotype of respondents, we designed the primers of XPC rs2228001 (forward: AGCAGCTTCCCACCTGTTC; reverse: GTGGGTGCCCCTCTAGTG); and XPC rs3731055 (forward: AGGCACGACTGGCCATTTT; reverse: AGGAGGTCGCTCGAAGGA) to run using the Roche LightCycler 96 real-time fluorescence quantitative PCR instrument. We mixed a 20-μl reaction system including 0.4-μl dNTP mixture, 0.4-μl 10-μM forward primer, 0.4 μl 10-μM reverse primer, 1.0-μl EvaGreen, 4.0-μl buffer, 1.0-μl DNA, and 12.8-μl H_2_O. The reaction condition was the same as the PCR amplification reaction, which started from 95°C for 600 s, and followed by 45 cycles of 95°C for 10 s, 60°C for 10 s, and 72°C for 15 s. The HRM curve analysis was performed using the accompanying Gene Scanning software version 1.1.0.1320 supplied with the LightCycler 96.

### Statistics

We used the Kolmogorov–Smirnov normality test to examine the normality of continuous variables. The concentrations of creatinine-adjusted urinary 1-OH-Pyr and the values of KRAS-2 and BRAF-15 were natural logarithm (ln) transformed to improve their normality. Normally continuous variables in this study were described using mean ± standard deviation (SD) and non-normal distributed variables were showed as medians with interquartile range (IQR) and categorical variables were presented as number (percentage). The concentrations of creatinine-adjusted urinary 1-OH-Pyr and the values of KRAS-2 and BRAF-15 were natural logarithm (ln) transformed because of the right-skewed distribution. The continuous variables of KRAS-2 and BRAF-15 were described as the dependent variable (*y*), respectively, in the multiple linear regression models with adjustment for working years (continuous), workplace (low exposure/high exposure), and smoking status (smokers/non-smokers) to estimate the association coefficients (β's) and their 95% confidence interval (95%CI) with per increment of creatinine-adjusted urinary 1-OH-Pyr. Age was excluded from the multiple linear regression models due to high correlation with working years (the Pearson coefficient *r* = 0.831; *p* < 0.001). Additionally, a restricted cubic spline model was employed to estimate the linear and non-linear shape of the associations of KRAS-2 and BRAF-15 with 1-OH-Pyr, respectively. The multiple linear regression models with adjustment of working years, smoking status, and workplaces were used to evaluate the effects of XPC genotype on DNA damage, accompanying with the relative β's and 95% CIs in individuals carrying rs2228001 GG/GT genotype combination with rs3731055 GG genotype, carrying rs2228001 GG/GT genotype or rs3731055 GG genotype against individuals carrying rs2228001 TT genotype combination with rs3731055 GA/AA genotype.

After 288 participants were further classified into three subgroups (T1, T2, and T3 subgroups) by the tertiles of 1-OH-Pyr, we employed the multiple linear regression models with adjustment of working years, workplace, and smoking status to calculate the *p*-trend values, with the relative β's and 95% CIs in T2 and T3 against T1 as the reference. Additionally, we also categorized all the study subjects into low (less than the 50th percentile of creatinine-adjusted 1-OH-Pyr) and high (above the 50th percentile of 1-OH-Pyr) 1-OH-Pyr subgroups. Hardy–Weinberg equilibrium (HWE) for the two SNPs was tested by a goodness-of-fit χ^2^-test before the analysis. Moreover, the joint effects of dichotomous 1-OH-Pyr (low and high exposure) with smoking status (smokers and non-smokers), XPC rs2228001 (TT, GG+GT) and XPC rs3731055 (GA+AA, GG) on KRAS-2 were further estimated using the multiple linear regression models with adjusting for working years and workplaces.

We conducted a restricted cubic spline model using the R software (version 3.4.1). The other data analyses with SPSS18.0 (SPSS Inc., Chicago, IL, USA). The Bonferroni-type correction was used for the multiple comparisons and *p* < 0.025 (after Bonferroni correction for two comparisons) was defined statistically significant. A two-sided *p* < 0.05 was considered as statistical significance for all other analysis.

## Results

### Subject characteristics

The general characteristics and the values of KRAS-2 and BRAF-15 for workers in the different internal exposure are showed in Table 1. No differences were observed in the distributions of age, working years, and smoking status among these three groups (all *p* > 0.05). In addition, after adjustment for smoking status, working years, and workplaces, we observed the high urinary 1-OH-Pyre group (median: 11.63) had significantly higher values of KRAS-2 and BRAF-15 than those in the intermediate 1-OH-Pyre group (median: 4.60) and low 1-OH-Pyre group (median: 2.42), respectively, (both *p* < 0.001, Table 1).

**Table 1:** General characteristics of study participants and exon genetic damage index stratified by tertiles of urinary 1-OH-Pyr levels.

**Variables**	**Overall (*****n*** = **288)**	**Low exposure** **(*****n*** = **95)**	**intermediate exposure** **(*****n*** = **97)**	**High exposure** **(*****n*** = **96)**	***p***
General characteristics					
Age (years, mean ± SD)	25.50 ± 2.97	25.27 ± 2.82	25.59 ± 2.73	25.32 ± 3.35	0.721^a^
Working years (years, mean ± SD)	3.00 ± 1.27	3.07 ± 1.22	3.14 ± 1.22	3.12 ± 1.37	0.917^a^
Smoking status (yes/no)	195/93	63/32	68/29	64/32	0.824^a^
Workplaces (coal preparation recovery workshop/coking oven workshop)	151/137	71/24	55/42	25/71	< 0.001^a^
urinary 1–OH–Pyr (median, 25th-75th percentile) (μmol/mmol creatine)	4.62 (2.93–8.47)	2.42 (1.84–2.92)	4.60 (4.12–5.13)	11.63 (8.37–19.72)	< 0.001^b^
Exon genetic damage index of gene					
KRAS−2 (median, 25th−75th percentile)	2.81 (2.47–3.15)	2.62 (2.31–2.89)	2.84 (2.56–3.19)	2.96 (2.62–3.39)	< 0.001^b^
BRAF−15 (median, 25th−75th percentile)	4.17 (3.67–4.67)	3.88 (3.41–4.39)	4.10 (3.66–4.52)	4.59 (4.13–5.21)	< 0.001^b^

1–OH–Pyr, 1–hydroxypyrene; KRAS−12, exon 12 genetic damage index of v–Ki–ras2 Kirsten rat sarcoma viral oncogene homolog gene; BRAF−15, exon 15 genetic damage index of v–raf murine sarcoma viral oncogene homolog B1 gene. Values shown are mean ± SD, n (%) and median (25th percentile, 75th percentile), or n (%).

a One–way ANOVA for continuous variables and Chi–squared test for categorical variables.

b Multivariate analysis of covariance with adjustment for smoking status, working years, and workplaces.

### Associations of urinary 1-OH-Pyr with damage index of exon 2 of KRAS and damage index of exon 15 of BRAF

**Figure 1:**
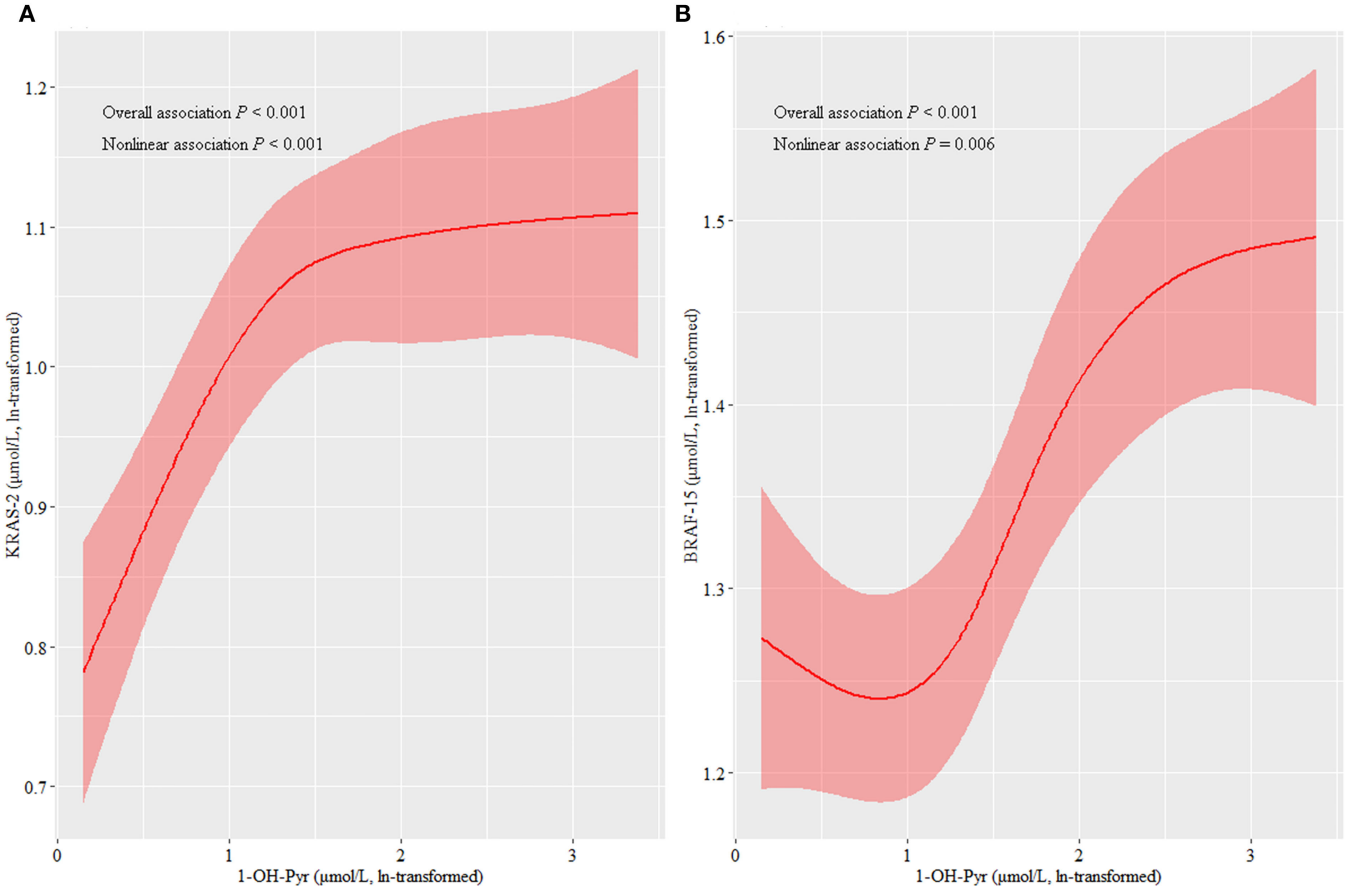
The associations of urinary 1-OH-Pyr with KRAS-2 and BRAF-15 based on the restricted cubic spline function using a smooth 5 default knots (5th, 27.5th, 50th, 72.5th, and 95th percentiles).

**Figure 2:**
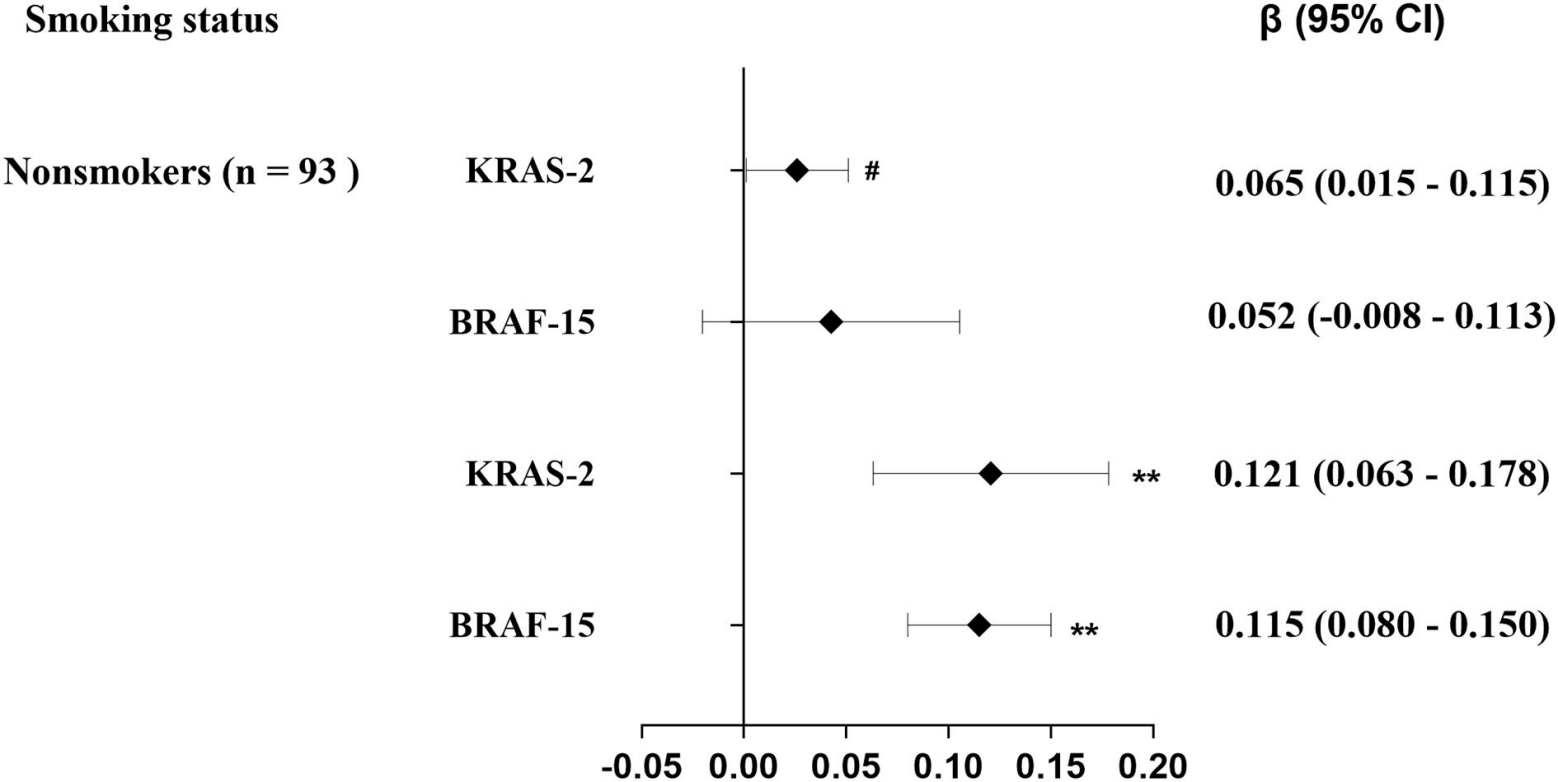
Stratification analysis of the estimated difference in exon genetic damage index [β (95% CI)] associated with a 1-SD increase in exposure levels of urinary1-OH-Pyr among 288 coke oven workers by smoking status. The lines in panels represent β (95% CI) based on multiple linear regression models adjusted for working years and workplace; ^#^*p* = 0.045 > 0.025; **p < 0.001.

As shown in Table 2, the median of KRAS-2 in tertiles of urinary 1-OH-Pyr levels was 2.62, 2.84, and 2.97; and 3.88, 4.10, and 4.59 for BRAF-15 in the first, second, and third urinary 1-OH-Pyr tertiles, respectively. The multiple linear regression models were used to estimate the associations of urinary 1-OH-Pyr with KRAS-2 and BRAF-15, and we observed both KRAS-2 and BRAF-15 were significantly gradually increased in subjects in the middle and upper tertiles of urinary 1-OH-Pyr compared to the subjects in the lower tertile of urinary 1-OH-Pyr after adjustment for smoking status (model 1) and smoking status, working years, and workplace (model 2) (all *p*_trend_ < 0.001). In addition, the adjusted β coefficients (95% CI) for ln-transformed KRAS-2 per increment of ln-transformed urinary 1-OH-Pyr were 0.103 (0.066–0.140) and 0.101 (0.061–0.141); 0.088 (0.059–0.117) and 0.087 (0.056–0.119) for ln-transformed BRAF-15 per increment of ln-transformed urinary 1-OH-Pyr in adjusted models 1 and 2, respectively (all *p* < 0.001). Furthermore, the multivariable-adjusted restricted cubic spline curve analyses showed the associations of urinary 1-OH-Pyr with KRAS-2 and BRAF-15 (*p* for non-linearity < 0.001 and *p* = 0.006, respectively), which confirmed the positive non-linear relationships (Figure 1). In stratified analyses, after adjustment for working years and workplaces, we found that the values of KRAS-2 and BRAF-15 only in smokers were positively associated with the concentrations of urinary 1-OH-Pyr with adjustment for working years and workplaces (both *p* < 0.001, Figure 2).

**Table 2:** The estimated difference in ln–transformed exon genetic damage index [β (95% CI)] associated with tertiles of urinary 1–OH–Pyr and per increment of ln–transformed urinary 1–OH–Pyr (n = 288).

**Variables**	**Tertile of urinary 1–OH–Pyr (**μ**mol/mmol creatine)**			***p–trend***	**Per increment of ln–transformed** **urinary 1–OH–Pyr**	
	**T**_1_ **(**< **3.07)**	**T**_2_ **(3.07–6.31)**	**T**_3_ **(**> **6.31)**		β **(95% CI)**	***p***
**KRAS−2**						
damage index of exon 12, Median	2.62	2.84	2.97		2.82	
No. of Participants	97	95	96		288	
Model 1^a^	0 (reference)	0.153 (0.075–0.230)	0.189 (0.111–0.267)	< 0.001	0.103 (0.066–0.140)	< 0.001
Model 2^b^	0 (reference)	0.149 (0.070–0.227)	0.178 (0.093–0.263)	< 0.001	0.101 (0.061–0.141)	< 0.001
**BRAF−15**						
damage index of exon 21, Median	3.88	4.10	4.59		4.20	
No. of Participants	97	95	96		288	
Model 1	0 (reference)	0.058 (−0.001–0.118)	0.198 (0.139–0.258)	< 0.001	0.088 (0.059–0.117)	< 0.001
Model 2	0 (reference)	0.060 (0.000–0.120)	0.200 (0.135–0.265)	< 0.001	0.087 (0.056–0.119)	< 0.001

a Model 1 was adjusted for smoking status.

b Model 2 was adjusted for smoking status, working years, and workplace.

### Effects of XPC SNPs on KRAS-2 and BRAF-15

**Figure 3:**
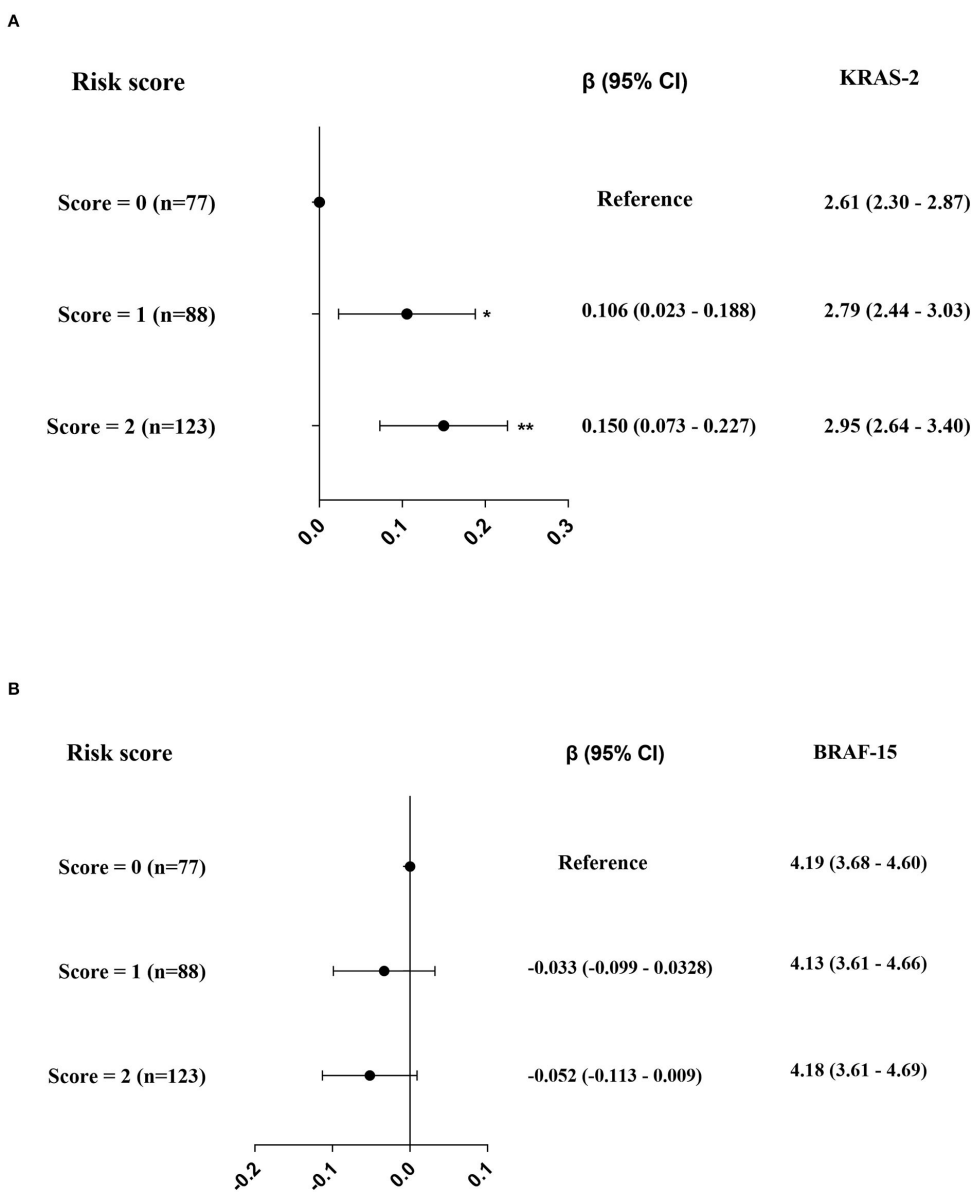
The effects of rs2228001 GG + GT genotype and/or rs3731055 GG genotype on KRAS-2 **(A)** and BRAF-15 **(B)** respectively. Note: The black solid dot and black line in panels represent a (95% CI), while the levels of KRAS-2 and BRAF-15 are represented as median (25th, 75th percentiles). ^*^p < 0.025; ^**^p < 0.001.

Genotype distribution of the two polymorphisms, rs2228001 and rs3731055, were in Hardy–Weinberg equilibrium (both *p* > 0.05). The associations of KRAS-2 and BRAF-15 with XPC genotypes in the 288 coke oven workers are listed in Table 3. We found that the rs2228001 minor allele (G-allele) is strongly associated with increased KRAS-2 (*p*_trend_ < 0.001), the medians of KRAS-2 in TT, TG, and GG genotypes were 2.65, 2.89, and 2.99, respectively. Additionally, the rs3731055 GG homozygote genotype was associated with higher KRAS-2 (*p*_trend_ = 0.006), the medians of KRAS-2 in GG, GA, and AA genotypes were 2.90, 2.63, and 2.76, respectively. We failed to observe the significant associations of BRAF-15 with rs2228001 and rs3731055 genotypes. In addition, we further assessed the effect of XPC genotypes on exon damage by sup-grouping 288 participants into rs2228001 G-allele combination with rs3731055 GG homozygote genotype defined as a risk score of 2 (score = 2) carriers, rs2228001 G-allele or rs3731055 GG homozygote genotype defined as a risk score of 1 (score = 1) carriers, and rs2228001 TT homozygote genotype combination with rs3731055 A-allele carriers defined as reference. Compared to the reference, we observed that 88 participants (30.6%) showed a significant increase effect (β = 0.106; *p* = 0.013) and 123 participants (42.7%) showed a significant increase effect (β = 0.150; *p* < 0.001) on KRAS-2 (Figure 3A). However, we failed to observe a significant impact of XPC SNPs on BRAF-15 (Figure 3B).

**Table 3:** Exon genetic damage index of gene according to genotypes.

**Variables**	**Major allele(A)**	**Minor allele(a)**	**N (AA/Aa/aa)**	**Exon genetic damage index of gene, median** **(25th, 75th percentiles)**			***p*** **–trend** ^*^
				**AA**	**Aa**	**aa**	
KRAS−12							
XPC							
rs2228001	T	G	110/145/33	2.65 (2.36–2.91)	2.89 (2.57–3.29)	2.99 (2.52–3.55)	< 0.001
rs3731055	G	A	156/112/20	2.90 (2.57–3.39)	2.63 (2.38–2.94)	2.76 (2.42–2.90)	0.006
BRAF−15							
XPC							
rs2228001	T	G	110/145/33	4.20 (3.68–4.61)	4.13 (3.60–4.65)	4.26 (3.63–5.06)	0.326
rs3731055	G	A	156/112/20	4.19 (3.63–4.68)	4.12 (3.64–4.61)	4.40 (3.87–4.79)	0.148

* Linear regression models with adjustment for smoking status, working years, and workplaces.

### Joint effects of smoking status, Rs2228001 genotypes, and Rs3731055 genotypes with urinary 1-OH-Py on KRAS-2

**Figure 4:**
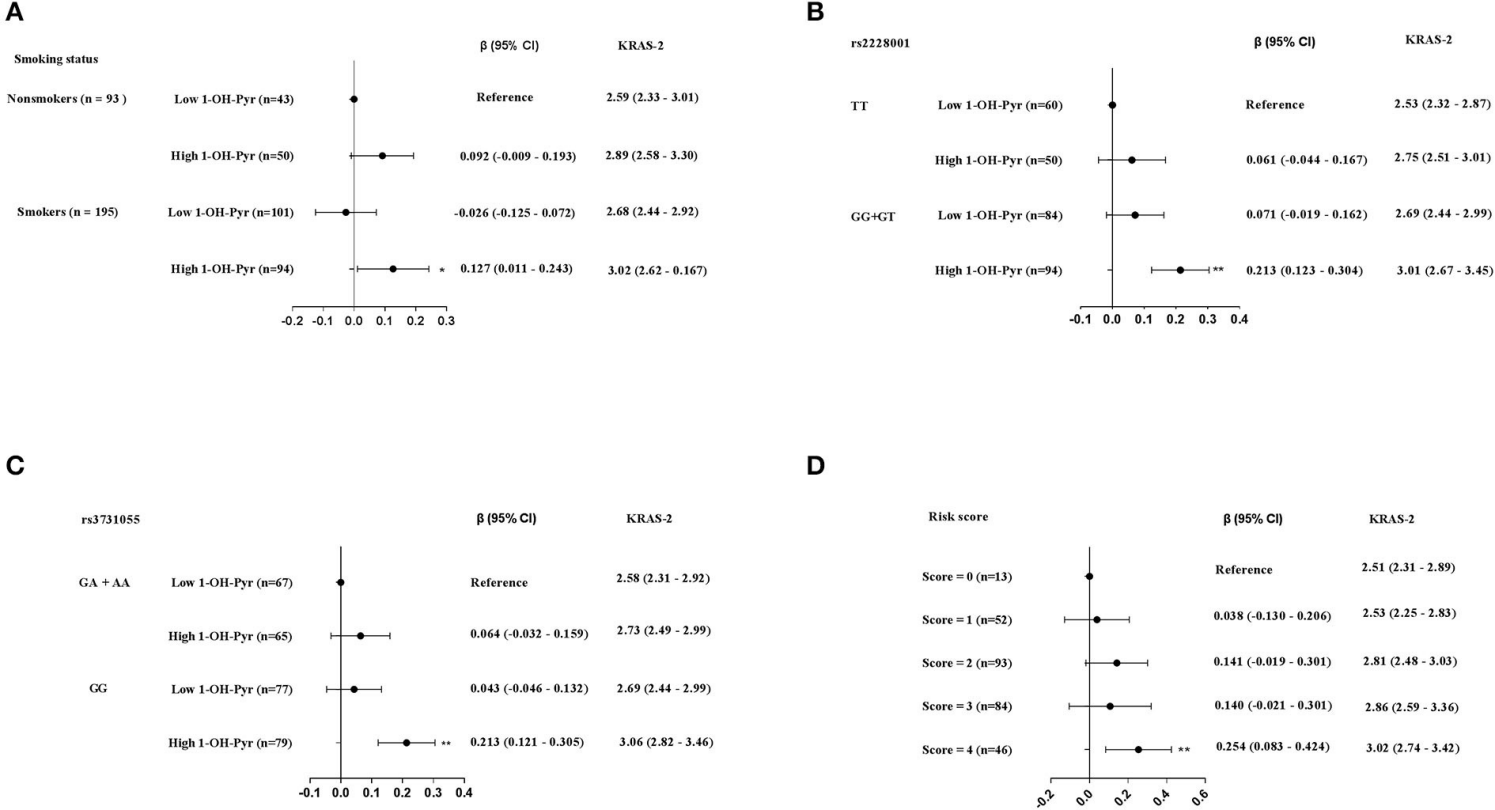
The joint effects of high level of urinary 1-OH-Pyr with **(A)** smoking status. **(B)** rs2228001 GG+GT genotype on KRAS-2. **(C)** rs3731055 GG genotype on KRAS-2. **(D)** The joint effects of the above four risk factors on KRAS-2. The black solid dot and black line in panels represent β (95% CI), while the KRAS-2 levels are represented as median (25th, 75th percentiles); ^**^p < 0.001.

After adjusted for working years and workplaces, we observed the non-smokers with high urinary 1-OH-Pyr showed the highest KRAS-2 (median: 3.02 *vs*. 2.59), with a 0.127 increase in values of KRAS-2 (95% CI: 0.011–0.243), compared to the non-smokers with low urinary 1-OH-Py (Figure 4A). Considering the small sample size and the strong effect of the rs2228001G-allele on KRAS-2, we then sub-grouped the 288 participants into rs2228001 TT and GG+GT genotype carriers and found the participants carrying rs2228001 GG+GT genotype had the highest KRAS-2 among the four subgroups with the rs2228001 TT genotype carriers as reference, who showed a 0.213 (95%CI: 0.123–0.304) increased KRAS-2 compared to the rs2228001 TT genotype carriers with low urinary 1-OH-Py (median KRAS-12: 3.01 *vs*. 2.53) (Figure 4B). In addition, given the weak effect of the rs3731055 A-allele compared to rs3731055 GG homozygote genotype on KRAS-2 as Table 3 showed above, the 288 participants were further categorized into rs3731055 GA+AA and rs3731055 GG genotype carriers. Compared with the rs3731055 GA+AA genotype carriers with low1-OH-Pyr, we observed the rs3731055 GG genotype carriers with urinary 1-OH-Pyr showed the highest level of KRAS-2 (median KRAS-12: 3.06 *vs*. 2.58), conferring a 0.213 increase in KRAS-2 (95% CI: 0.121–0.305) (Figure 4C).

Furthermore, we then explored the joint effects of the aforementioned four risk factors including smoking, carrying rs2228001 GG+GT genotype, carrying rs 3731055 GG genotype and high urinary 1-OH-Py. Compared to these participants without any risk factors (as reference), we observed that 46 participants (16%) carrying 4 risk factors showed significant higher KRAS-2 (median KRAS-2: 3.02 *vs*. 2.51), and the adjusted β coefficients with 95% CI from regression models adjusted for working years and workplaces were 0.254 (0.083–0.424) (Figure 4D).

## Discussion

In this study, we found PAHs exposure were positively associated with both KRAS-2 and BRAF-15, respectively, following non-linear dose-response pattern among 288 coke oven workers. Then associations of urinary 1-OH-Pyr with KRAS-2 were observed among both smokers and non-smokers, and the adjusted β coefficients were stronger among smokers than that among non-smokers. Nevertheless, the significant associations between1-OH-Pyr and BRAF-15 were found only in smokers. The subsequent analyses indicated that XPC genetic polymorphism, marked by SNP rs2221008 and rs3731055, were significantly associated with KRAS-2 and this associations may be modulated by 1-OH-Pyr levels. More importantly, we revealed the joint effects of PAHs, smoking and XPC genetic polymorphism on increasing KRAS-2.

The PAHs' exposure is a crucial public health concern worldwide because of their genotoxic and carcinogenic properties and associated with DNA damage and increased risk of developing lung cancer. In this study, we used urinary 1-OH-Pyr as suitable and sensitive biomarker to reflect internal PAHs exposure.

The oncogene mutation KRAS is one of the key driver mutations in NSCLC (29), and approximately 97% of KRAS mutations in NSCLC involve codons 12 or 13 in exon 2 (30). Mutations in BRAF, observed in 2–4% of NSCLCs, mainly occur transversion of thymidine to adenosine at nucleotide T1799A on exon 15, also existing the mutation of G469A and D594G in BRAF (31). The latest evidence has shown that DNA damage plays an important role in the DNA mutational signatures (32). In this study, we observed a linear dose–response relationship between urinary 1-OH-Pyr and damage in exon 2 of KRAS and damage in exon 15 of BRAF. The previous evidence has also proved that PAHs in smoky coal emissions can induce genetic mutations in KRAS genes and the mutation in KRAS gene can reflect the PAH exposure (19). Also, two investigations showed that KRAS mutations were associated with exposure to smoky coal; based on the mutation spectra in tumor genes, the gene mutation can be attributed to direct DNA damage from mutagenic exposures (19, 33). Our result was consistent with the reported study in which point mutation in KRAS gene was represented the PAH exposure in mice (34).

Smoking is a major environmental risk factor contributing to DNA damage. Especially, it is proved that smoking is an independent factor for KRAS mutation in NSCLC (35). One of the characteristics of NSCLC in smokers is the DNA damage effect by tobacco carcinogens, including PAHs. It has been identified that most of the driver gene alterations in lung adenocarcinoma in never-smokers include EGFR, KRAS mutations, and so on (36). In this study, we also explore the associations of urinary 1-OH-Pyr with damage in exon 2 of KRAS and damage in exon 15 of BRAF in smokers and non-smokers, and found significant positive associations of urinary 1-OH-Pyr with damage in exon 2 of KRAS in both smokers and non-smokers, but a stronger effect was observed in smokers compared to that in non-smokers, suggesting tobacco smoking is a contributor to damage in exon 2 of KRAS. Additionally, we found the significant positive association between urinary 1-OH-Pyr and damage in exon 15 of BRAF in smokers but not in non-smokers. Unlike EGFR mutation, which is increased in never smokers, KRAS mutation in NSCLC has an odd decrease among never smokers (29), and it is typically found in tumors from patients who smoke (often heavy smokers) (18). Also, BRAF mutation, another different lung cancer driver mutation, is frequent in smoking patients (37). These previous evidences, along with the results from this study, demonstrated that damage in exon 2 of KRAS and damage in exon 15 of BRAF could be served as novel biomarkers for DNA damage and maybe as potential mediators for carcinogenesis induced by PAHs exposure and cigarette smoking.

The genetic variant XPC, as an important protein in the NER pathway, plays a crucial role in modulating the effects on repairing damaged DNA from the environmental exposure to maintain the genetic integrity (38, 39). This study was further intended to investigate whether both XPC rs2221008 and rs3731055 influence the susceptibility of damage in exons in KRAS and BRAF induced by the combined exposure to PAHs and smoking, and illustrated that individuals carrying XPC rs2228001G allele were at a significantly increased risk for damage in exon 2 of KRAS, and carriers of the rs3731055 GG homozygote genotype were associated with higher damage in exon 2 of KRAS. Evidence shows that XPC polymorphisms are associated with the different capacity to repair DNA damage and further impact the individual's susceptibility to lung cancer (40). Similar research has proved that the carriers of the XPC rs2228001 and the XPC rs3731055 are related with DNA damage levels in coke oven workers (24). These risk factors including cigarette smoking, high urinary 1-OH-Pyr, carrying rs2228001G allele and carrying 3731055 GG homozygote genotype, were simultaneously considered to explore their joint effect on damage in exon 2 of KRAS. And we observed that only a small minority of participants (16.0%) with all four risk factors had significant joint effect on damage in exon 2 of KRAS, indicating XPC genetic effects on damage in exon 2 of KRAS are stronger in cigarette smokers with higher exposure to PAHs than in non-smokers with lower exposure to PAHs, which provides us useful information on the role of co-exposure to PAHs and smoking in inducing damage in exons, and XPC genetic polymorphism may partly confers increased susceptibility of individuals to damage in exon of KRAS associated with combined exposure to PAHs and cigarette smoking, as well as strategies should be designed to protect this subpopulation with these risk factors.

This study certainly has some major strengths. This study is population-based design with a high participation rate (> 97%), and we detected urinary 1-OH-Pyr, a sensitive biomarker to evaluate the individual PAHs exposure levels, and the levels of damage in exons in KRAS and BRAF genes, in particular, KRAS genes are viewed as critical DNA targets for environmental carcinogens. In addition, considering XPC gene plays an important role in the initiation of DNA repair, we further investigated whether XPC genetic polymorphisms regulated the effects of PAHs exposure on exon damage in individuals with regular exposure to coke oven emission rich in PAHs at least 3 months. Our findings showed that joint effects of PAHs exposure with the well-known risk factors, such as cigarette smoking regulated by genetic variation on exon damage levels, are in line with previous findings, which could provide scientific evidence to develop corresponding protective intervention for susceptible population. However, this study is a cross-sectional and exploratory design in which our results are difficult to establish a causal relationship between co-exposure to PAHs and smoking, XPC genetic polymorphisms, and damage in exons. Further functional studies are warranted to elucidate the underlying the molecular mechanisms, and we plan to conduct further biochemical studies and functional studies to elucidate the biological plausibility in this study. Additionally, given the small sample size and this study was carried out among occupational population only aged 19–35 years following the inclusion and exclusion criteria strictly, whether our findings can be extrapolated to the general population remains to be explored in further research with larger sample size.

## Conclusion

The findings in this study indicated that individuals with the XPC genetic variants (marked by rs2221008 G allele and rs3731055 GG homozygote genotype) may predict the susceptibility to damage in exon 2 of KRAS induced by PAHs from occupational exposure and cigarette smoking, which lend further insight to potential joint effects of genetic and environmental factors affecting lung carcinogenesis, as well as make it possible to provide evidence-based personalized prevention and intervention for deleterious health effects caused by environmental exposure.

## Data availability statement

The raw data supporting the conclusions of this article will be made available by the authors, without undue reservation.

## Ethics statement

The studies involving human participants were reviewed and approved by the Ethics Committee of Guangzhou Medical University approved the study (KY01-2019-02-09). The patients/participants provided their written informed consent to participate in this study.

## Author contributions

JL, SH, MY, XL, QH, YH, XY, and SC collected the samples and established the database and conducted the experiments. MY, YH, and BY designed and carried out the study. SC, XY, and BY conducted the data analysis and drafted the manuscript. BY revised the manuscript. All the authors had access to the data and reviewed and approved the final submitted manuscript.

## Funding

This work was supported by the National Natural Science Foundation of China (Grant Nos. 81903381, 82160607, and 82173609), the Guangdong Province (Grant No. 2018A030313606), and Guangzhou Key Laboratory Fund (201905010004).

## Conflict of interest

The authors declare that the research was conducted in the absence of any commercial or financial relationships that could be construed as a potential conflict of interest.

## Publisher's note

All claims expressed in this article are solely those of the authors and do not necessarily represent those of their affiliated organizations, or those of the publisher, the editors and the reviewers. Any product that may be evaluated in this article, or claim that may be made by its manufacturer, is not guaranteed or endorsed by the publisher.
